# Angioleiomyoma of Broad Ligament

**DOI:** 10.4103/0974-1216.71609

**Published:** 2009

**Authors:** S Agarwal, S K Gupta, N Tejwani

**Affiliations:** Department of Pathology, Lady Hardinge Medical College, New Delhi, India

**Keywords:** Angioleiomyoma, broad ligament, capillary

## Abstract

Angioleiomyoma is an uncommon benign mesenchymal neoplasm that originates from smooth muscle cells and contains numerous thick-walled blood vessels. Here, we are presenting a case report of a huge broad ligament angioleiomyoma because of its rarity.

## INTRODUCTION

Angioleiomyoma is an uncommon benign mesenchymal neoplasm that originates from smooth muscle cells and contains numerous thick-walled blood vessels. It is usually found in the skin of lower extremities but is extremely rare in broad ligament.[1]

## CASE REPORT

A 45-year-old multiparous female presented with abdominal pain and lump since 2 months. No history of menstrual irregularities was noted. Sonography (USG) and Contrast Enhanced Computerized Tomography (CECT) showed a large homogeneous solid mass in the lower abdomen. The mass was hypodense in attenuation and showed mottled heterogeneous enhancement.

Pre-operatively, the mass was seen arising from right broad ligament, deriving its blood supply from omental blood vessel. However, uterus and bilateral adnexa were normal and separate from the tumor. Mild ascitis was also seen. Only the tumor excision was performed.

### Pathologic findings

We received a single large encapsulated mass measuring 25 × 10 × 10 cm for histopathologic examination. External surface was smooth and congested, and dilated blood vessels seen. Cut surface showed predominantly solid mass, gray white in color, having multiple dilated and congested blood vessels, giving it a multicystic appearance.

### Microscopic examination

Hematoxylin and eosin stained section showed well-encapsulated mass comprising spindle-shaped smooth muscle cells arranged in fascicles intermingled with many thick-walled dilated and congested blood vessels. The inner layer of vessel wall was arranged in a circumferential fashion and the outer layer of these smooth muscle cells swirled away from vessel wall merging with surrounding tissue. No mitotic figure and necrosis was observed. Areas of myxoid and hyaline degeneration were also present.

Van Gieson’s elastic stain (VGE) showed disruption in internal elastic lamina of thick-walled blood vessels. On immunohistochemistry, these tumor cells were positive for SMA, CD31 and CD34, thus confirming the diagnosis of angiolieomyoma.

## DISCUSSION

Angioleiomyoma is a benign mesenchymal neoplasm consisting of smooth muscle cells and thick-walled blood vessels commonly found in the skin of extremities and rarely occurs in uterus.[1] In the series reported by Hachisuga *et al*, 375 of 562 occurred in the lower extremities, 125 in the upper extremities, 48 on the head and 14 on the trunk. Most of these cases were less than 2 cm in diameter.[2] There are only a few cases reported in head and neck region and even in submandibular glands.[3,4] In the head and neck region, this lesion presented as small and painless lump.[3] However, this tumor always presented as painful lesion when located in uterus as seen in our case. It is more common in females and usually occurs between fourth and sixth decades of life. Although the exact mechanism of pain remains unknown, it is thought to be related to local ischemia because of vascular contraction.[3]

The tumor on gross appearence is whorled dilated blood vessels, giving it a multicystic appearance,[5] as seen in our case, wherein it was huge, single, well-encapsulated mass with congested and dilated blood vessels. Microscopically, this tumor composed of smooth muscle cells with thick-walled blood vessels.

Histologically, angioleiomyomas are described into three subtypes: Capillary or solid, cavernous, venous or mixed. Our case was of capillary subtype.

Immunohistochemical markers for smooth muscle cells such as smooth muscle actin (SMA) and vessel marker CD34 and CD31 are essential to differentiate angioleiomyoma from other tumors such as angiofibroma, angiomyolipoma and angiomyfibroblastoma. All these tumors are positive for vimentn and desmin but negative for smooth muscle actin.[6] Our case was positive for smooth muscle actin, CD 34, CD 31, and negative for vimentin and desmin. Hence, the diagnosis of angioleiomyoma was confirmed. Moreover, our case also showed disruption of internal elastic lamina on VGE stain.

Degenerative changes in the angioleiomyomas are due to ischemia and the types of degenerative changes depend on the degree and rapidity of the onset of vascular insufficiency. Hyaline changes are the commonest form of degeneration. We present this case because of its extreme rarity and large size of the tumor.

Treatment of choice is tumor excision open or laparoscopic, as none of the cases reported by Duhig and Ayer[7] developed recurrence following excision. Till date, the follow-up of our patient has also been uneventful. Repeat computed tomography (CT) scan did not reveal any evidence of recurrence.
